# Unveiling the complete organelle genomes of *Gypsophila vaccaria: de novo* assembly and evolutionary insights into a medicinally important species

**DOI:** 10.3389/fpls.2025.1684062

**Published:** 2025-10-01

**Authors:** Chaoqiang Zhang, Ruifeng Yang, Mengyue Wang, Jiayin Zhang, Jingting Shen, Bin Yang, Dongzhi Zhang, Liang Yin, Xiaoming Wang, Chien-Hsun Huang, Jinglong Li

**Affiliations:** ^1^ Key Laboratory of Hexi Corridor Resources Utilization of Gansu, College of Life Sciences and Engineering, Hexi University, Zhangye, Gansu, China; ^2^ State Key Laboratory of Reproductive Regulation and Breeding of Grassland Livestock, School of Life Sciences, Inner Mongolia University, Hohhot, Inner Mongolia, China; ^3^ Key Laboratory of Herbage and Endemic Crop Biology, Ministry of Education, School of Life Sciences, Inner Mongolia University, Hohhot, Inner Mongolia, China; ^4^ Ministry of Education Key Laboratory for Biodiversity Science and Ecological Engineering, Institute of Biodiversity Science, Fudan University, Shanghai, China; ^5^ Shanghai Ninth People’s Hospital, Shanghai Jiao Tong University School of Medicine, Shanghai, China; ^6^ College of Agriculture and Ecological Engineering, Hexi University, Zhangye, Gansu, China

**Keywords:** *Gypsophila vaccaria*, mitochondrial genome, chloroplast genome, phylogenetic analysis, RNA editing events

## Abstract

**Introduction:**

*Gypsophila vaccaria* (Caryophyllaceae) is a medicinal plant with over 2,000 years of documented use in China. Despite its known pharmacological properties and phytochemical profile, no organellar genomic resources are currently available, limiting evolutionary studies and molecular breeding efforts.

**Methods:**

We assembled the complete mitochondrial (361,814 bp) and quadripartite chloroplast (150,050 bp) genomes of *G. vaccaria* using HiFi sequencing. Codon usage, RNA editing, and selection pressure were analyzed, and phylogenomic relationships were inferred. Species-specific SSR markers were identified for potential molecular applications.

**Results:**

HiFi-based assembly revealed exceptional mitochondrial genome plasticity, with 15.6% (56.7 Kb) derived from chloroplast DNA transfers—the highest reported in Caryophyllaceae—including 12 functional genes (e.g., rps7, ndhB, rrn16S). Both organellar genomes show A/U-biased codon usage (mitochondrial RSCU: 29/44 codons) and divergent RNA editing (257 mitochondrial vs. 105 chloroplast C-to-U sites). Positive selection (Ka/Ks > 1) was detected in cytochrome c maturation genes (ccmFN, ccmB, ccmFC), contrasting with overall purifying selection (median ω = 0.32). Phylogenomic analyses robustly resolved Caryophyllaceae–Amaranthaceae sisterhood (BS = 100%).

**Discussion:**

As the first organellar genomes from Gypsophila, this study provides insights into lineage-specific adaptations through chloroplast-mitochondrial co-evolution. The 56.7 Kb MTPTs and positively selected cytochrome c genes serve as targets for adaptive evolution research, while 81 species-specific SSRs facilitate molecular marker development in Caryophyllaceae.

## 1 Introduction

*Gypsophila vaccaria* Sm. (Caryophyllaceae) is an herbaceous plant native to temperate regions of Asia, Europe and other parts of the world. In China, it is widely distributed except in the southern regions (Tian et al., 2021). The dried, mature seeds of *G. vaccaria*, commonly known as “Wang Bu Liu Xing”, have long been used in traditional Chinese medicine (TCM) to treat amenorrhea, dysmenorrhea, mastitis, and urinary diseases (Sang et al., 2003; Committee, 2020; Tian et al., 2021), as well as to promote diuresis and milk secretion and to relieve carbuncles (Sang et al., 2003). As documented in the oldest materia medica, Shen Nong Ben Cao Jing, *G. vaccaria* has been used in China for 2000 years (Tian et al., 2021). In recent years, its clinical applications have expanded, with new uses identified, such as the treatment of gallstones (You, 1989), shingles (Tan, 2006), rhinitis (Xing and Jin, 2013), benign prostatic hyperplasia (Qi et al., 2013; Zhang et al., 2013), and hypertension (Liu, 2018). Phytochemical studies have revealed that flavonoids, triterpenoid saponins, cyclic peptides, and polysaccharides are the main bioactive components of G. vaccaria seeds (Zhou et al., 2016; Tian et al., 2021). These compounds exhibit vasodilatory, anticoagulant, anti-inflammatory, antitumor, antiangiogenic, and antioxidant activities, as well as estrogen-like effects (Li and Liang, 2007; Hu et al., 2014; Tian et al., 2021; Bing et al., 2024). Additionally, they have been shown to alleviate osteoporosis and promote vasodilation (Hu et al., 2014). Overall, G. vaccaria has attracted increasing attention in recent years.

To date, studies on this species have primarily focused on its chemical constituents (Zhou et al., 2016) and pharmacological properties (Tian et al., 2021; Li N et al., 2022). However, no studies have reported its mitochondrial or chloroplast genomes, significantly limiting further research. In angiosperms, the nuclear genome is biparentally inherited, whereas chloroplasts and mitochondrial DNA are maternally inherited (Cheng et al., 2021; Tian et al., 2021). This uniparental inheritance pattern, which excludes paternal genetic contributions, serves as a powerful tool for investigating species origin, genetic diversity, classification, and phylogenetic relationships (Birky, 2001; Sibbald et al., 2021; Fan et al., 2022). The mitogenomes of Angiosperm display substantial size variation, spanning from 22 Kilobases (Kb) in *Avicennia marina* (Friis et al., 2021) to 11.7 Megabases (Mb) in *Larix sibirica* (Putintseva et al., 2020), although most species harboring genomes between 200 and 750 Kb. Most mitogenomes contain 19 to 64 known genes (excluding duplicate genes and ORFs), 5 to 25 introns, and highly variable intergenic regions (Burger et al., 2003; Bi et al., 2022). The *in vivo* conformation of plant mitogenomes displays greater diversity. While most assembled plant mitogenomes are circular, polycyclic structures have been reported in maize (Fauron et al., 1995) and kiwifruit (Wang et al., 2019), linear structures in Thuja sutchuenensis (Xia et al., 2023), and multi-branched structures in Punica granatum (Feng et al., 2023; Lu et al., 2023). This structural complexity makes mitogenome assembly challenging, resulting in significantly fewer studies compared to chloroplast and plastid genomes. As of May 2025, the NCBI Organelle Genome Database (https://www.ncbi.nlm.nih.gov/genome/browse#!/organelles/) identified 688 mitogenomes, 15,396 chloroplast genomes, and 1,718 plastid genomes, highlighting the urgent need for expanded mitogenomic investigations. Recent work has demonstrated that mitochondrial genomes can provide unique insights for medicinal species. In Dendrobium and Salvia, structural variation and recombinogenic repeats have yielded diagnostic markers useful for authentication (Yang et al., 2022; Tong et al., 2024). RNA editing in plant mitochondrial genome plays a role in energy metabolism and stress responses, potentially influencing the accumulation of pharmacologically active compounds (Li et al., 2024). These findings highlight the dual importance of mitogenomes for both basic and applied research in medicinal plants. Given its long history of medicinal use and growing clinical applications, *G. vaccaria* represents a valuable yet genomically neglected taxon. The absence of organellar genomic data, particularly the mitochondrial genome, presents a major knowledge gap. Therefore, assembling and annotating the complete mitogenome of *G. vaccaria* is critical.

In this study, we assembled and annotated the complete *G.vaccaria* mitogenome for the first time based on PacBio HiFi long-read sequencing data. We also assembled its chloroplast genome from the same dataset. Characteristics of the *G.vaccaria* mitochondrial genome and chloroplast genome were analyzed, including codon preference, RNA editing events, repetitive sequence analysis, Ka/Ks analysis, phylogenetic analysis, and sequence migration analysis. These results will provide a foundation for understanding the evolution, molecular breeding, structural characteristics and organelle**s** inheritance in *Gypsophila* species.

## 2 Results

### 2.1 Characteristics of the complete organelle genomes of *G. vaccaria*

**Figure 1:**
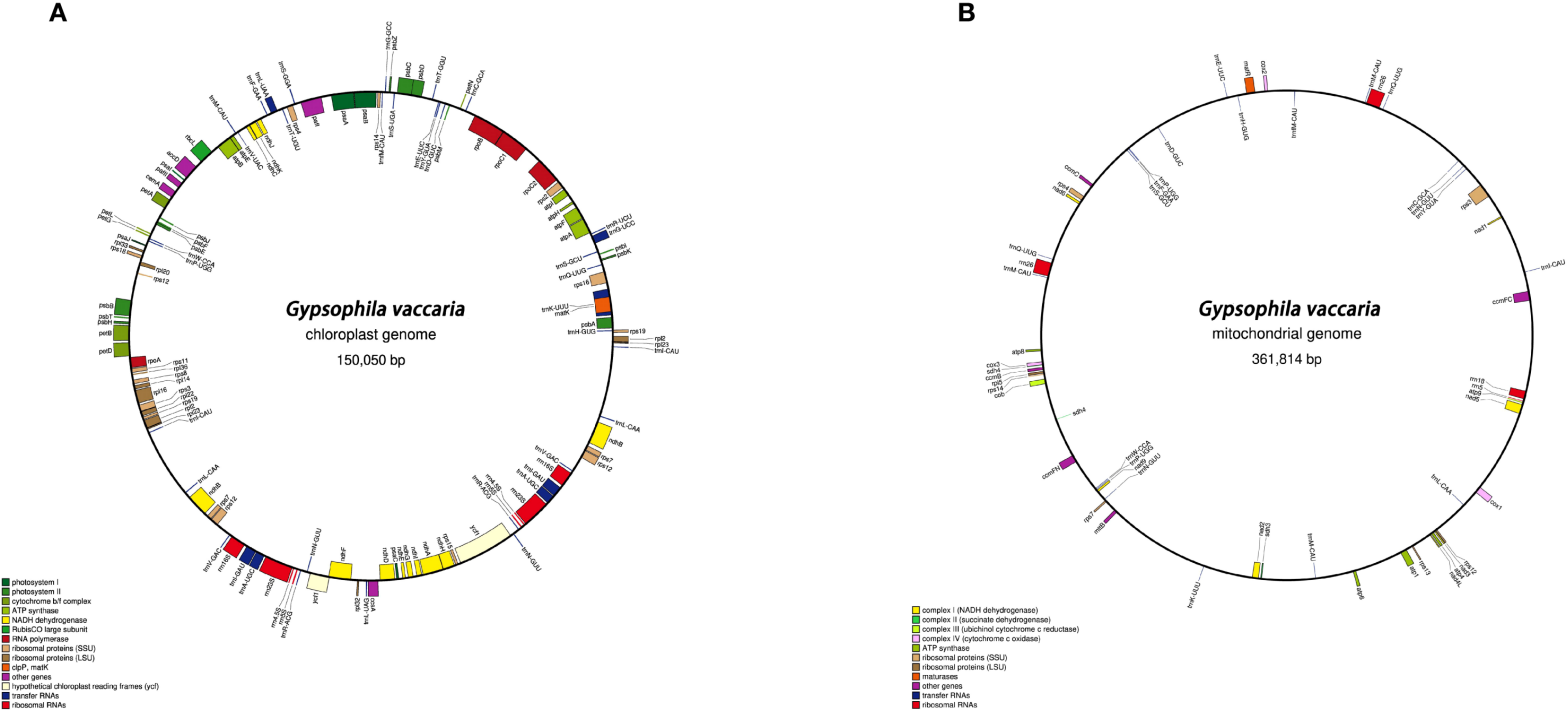
**(A)** Chloroplast genome of *G.vaccaria*. **(B)** Mitochondrial genome of of *G.vaccaria*. Genes are color-coded based on their functional groups. GC content is represented on the inner circle by the dark gray plot.

HiFi reads unambiguously resolved the chloroplast genome as a circular quadripartite molecule (150,050 bp; **Figure 1A**), with IR regions (25,167 bp each), a large single copy (LSC) region (82,717 bp), and a small single copy (SSC) region (16,999 bp) (**Figure 1A**). A total of 121 functional genes were annotated, including 77 protein-coding genes, 37 tRNAs, and 8 rRNAs (**Table 1**). Intron analysis identified 16 genes (e.g., *trnK-UUU*、*rps16*、*trnS-CGA*、*atpF*、*rpoC1*、*trnL-UAA*、*trnV-UAC*、*rpl16*、*ndhB*、*trnE-UUC*、*trnA-UGC*、*ycf1*、*ndhA*、*trnA-UGC*、*trnE-UUC* and *ndhB*) containing single intron, while two genes (*ycf3* and *ycf1*) possessed two introns each (**Supplementary Table S1**).

**Table 1:** Gene composition in the chloroplast genome of *Gypsophila vaccaria* plants.

Category of genes	Group of genes	Gene name
	tRNA	trnH-GUG,trnK-UUU,trnQ-UUG,trnS-GCU,trnS-CGA,trnR-UCU,trnC-GCA,trnD-GUC,trnY-GUA,trnE-UUC,trnT-GGU,trnS-UGA,trnG-GCC,trnM-CAU*,trnS-GGA,trnT-UGU,trnL-UAA,trnF-GAA,trnV-UAC,trnM-CAU*,trnW-CCA,trnP-UGG,trnL-CAA*,trnV-GAC*,trnE-UUC*,trnA-UGC*, trnR-ACG*, trnN-GUU*, trnL-UAG,
	rRNA	rrn16s*,rrn23s*,rrn4.5s*,rrn5s*
Genes for photosynthesis	Subunits of ATP synthase	*atpA, atpB, atpE, atpF, atpH, atpI*
	Subunits of photosystem II	*psbA, psbB, psbC, psbD, psbE, psbF, psbI, psbJ, psbK, psbM, psbN, psbT, psbZ, ycf3*
	Subunits of NADH-dehydrogenase	ndhA, ndhB*, ndhC, ndhD, ndhE, ndhF, ndhG, ndhH, ndhI, ndhJ, ndhK
	Subunits of cytochrome b/f complex	*petA, petB, petD, petG, petL, petN*
	Subunits of photosystem I	*psaA, psaB, psaC, psaI, psaJ*
	Subunit of rubisco	*rbcL*
Self replication	Large subunit of ribosome	rpl14, rpl16, rpl2*, rpl20, rpl22, rpl23*, rpl32*, rpl33, rpl36
	DNA dependent RNA polymerase	*rpoA, rpoB, rpoC1, rpoC2*
	Small subunit of ribosome	rps11, rps14, rps15, rps16, rps18, rps19*, rps2, rps3, rps4, rps7*, rps8
Other genes	Subunit of Acetyl-CoA-carboxylase	*accD*
	c-type cytochrom synthesis gene	*ccsA*
	Envelop membrane protein	*cemA*
	Maturase	*matK*
Unkown	Conserved open reading frames	ycf1, ycf1, ycf2*, ycf4

*: Number of copies of multi-copy genes

The complete mitochondrial genome of *G.vaccaria* is a circular DNA molecule with a total length of 361,814 bp. (**Figure 1B**). Nucleotide composition analysis revealed a total GC content of 43.82%, comprising 28.17% (A), 28.00% (T), 21.93% (C), and 21.88% (G). A total of 60 genes were annotated in the *G. vaccaria* mitogenome (**Figure 1B**; **Table 2**), including 35 protein-coding genes (PCGs: 28 core genes and 7 variable genes), 21 tRNA genes (4 multicopy), and 4 rRNA genes (1 multicopy) (**Table 2**). Interestingly, the *nad7* and *sdh4* genes were present in two copies, while one rRNA gene (*rrn26*) and four tRNA genes (*trnM-CAU*, *trnN-GUU*, *trnP-UUG trnQ-UUG*) exhibited either two or three copies. Among the annotated genes in *G. vaccaria*, seven contained introns: *ccmFC*, *nad1*, *nad2*, *nad4*, *nad5*, *nad7*, and *rps3*. (**Table 2**; **Supplementary Table S2**).

**Table 2:** Genetic composition of mitochondrial genome of the *G. vaccaria*.

Group of genes	Genes name
ATP synthase	*atp1, atp4, atp6, atp8, atp9*
Cytohrome c biogenesis	*ccmB, ccmC, ccmFC(1), ccmFN*
Ubichinol cytochrome c reductase	*cob*
Cytochrome c oxidase	*cox1, cox2, cox3*
Maturases	*matR*
Transport membrane protein	*mttB*
NADH dehydrogenase	nad1(4), nad2(4), nad3, nad4(2), nad4L, nad5(4), nad6, nad7(4)*, nad9
Subunit of succinate dehydrogenase	sdh3, sdh4*
Ribosomal proteins (LSU)	*rpl5*
Ribosomal proteins (SSU)	*rps12, rps13, rps14, rps3(1), rps4, rps7*
Ribosomal RNA	rrn18, rrn26*, rrn5
Transfer RNAs	trnM-CAU**, trnC-GCA, trnD-GUC, trnE-UUC, trnF-GAA, trnH-GUG, trnK-UUU, trnL-CAA, trnI-CAU, trnfM-CAU, trnN-GUU*, trnP-UGG*, trnQ-UUG*, trnS-GCU, trnW-CCA, trnY-GUA

*: Number of copies of multi-copy genes; Gene (number): intron number

### 2.2 Repeat sequences analysis in the G. vaccaria organelle genomes

**Figure 2:**
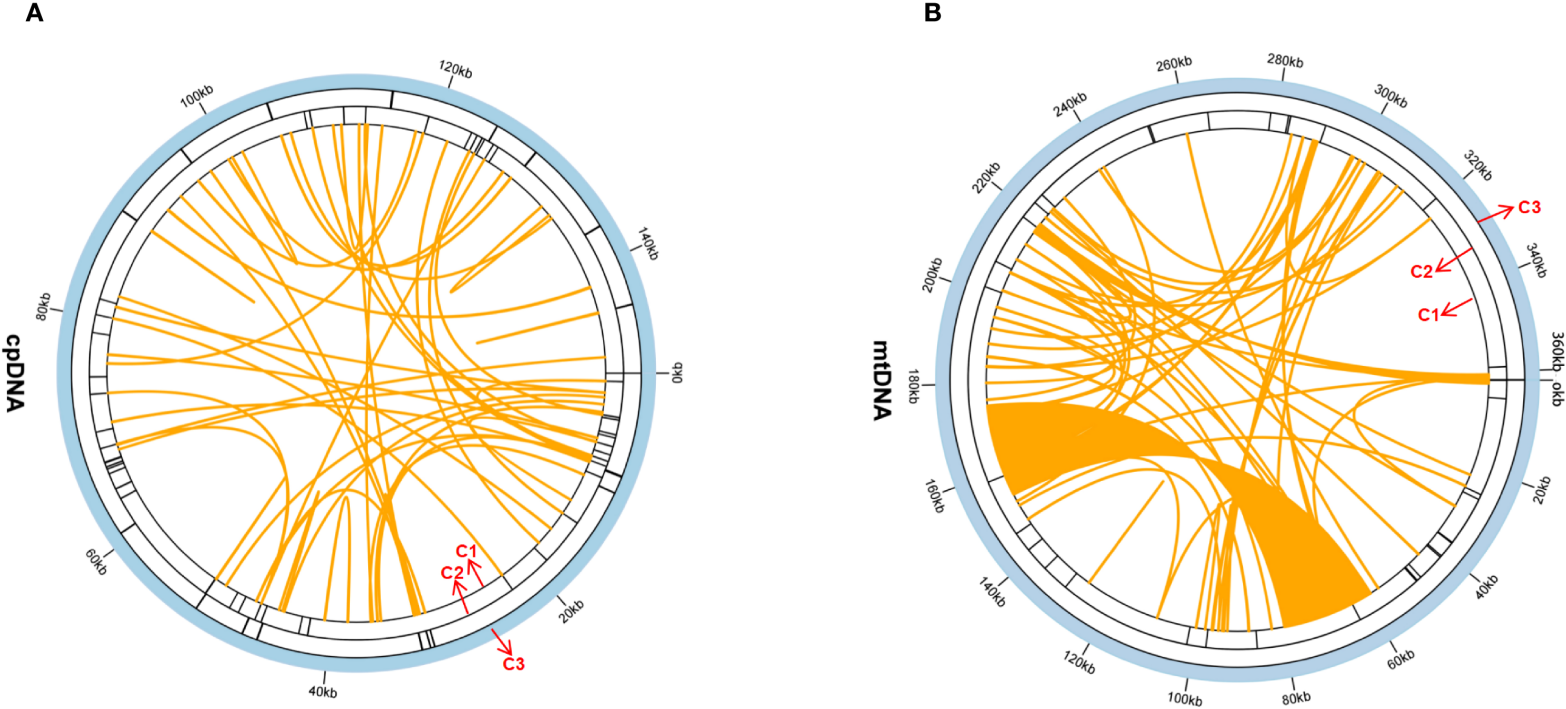
The repeat analysis of the *G.vaccaria* chloroplast genomes. **(A)** The repeat sequences identified in the chloroplast genome. **(B)** The repeat sequences identified in the mitochondrial genome. The C1 circle shows the dispersed repeats connected with yellow, blue, purple, and pink arcs from the center going outward. The C2 circle shows the tandem repeats as short bars. The C3 circle shows the microsatellite sequences identified using MISA. The scale is shown on the C3 circle, with intervals of 20 Kb.

In this study, 47 SSRs were detected in the chloroplast genome, whereas 34 in the mitochondrial genome (**Figure 2**; **Supplementary Tables S3**, **Supplementary Tables S4**). In the chloroplast genome, mononucleotide repeats—particularly A/T units—were the most abundant SSR type, representing 85.1% of the total. In the mitochondrial genome, they were similarly predominant (88.2%), closely matching the chloroplast pattern. Notably, the mitochondrial genome contained a trinucleotide repeat (TTC) that was undetected in the chloroplast genome. this TTC repeat could serve as a potential marker for identifying *G. vaccaria*.

Twenty-two tandem repeats (TRs) were detected in the chloroplast genome and 15 in the mitochondrial genome. Most TRs are located in intergenic regions (chloroplast: 18/22; mitochondrial: 12/15), with minor occurrences in coding regions (chloroplast: 3/22; mitochondrial: 2/15) and pseudogenes (chloroplast: 1/22) (**Figure 2**; **Supplementary Tables S5**, **Supplementary Tables S6**).

Surprisingly, only forward repeats were detected in both chloroplast and mitochondrial genomes (**Figure 2**, **Supplementary Tables S7**, **Supplementary Tables S8**). The absence of other repeat types suggests distinct evolutionary constraints on repeat-driven genomic plasticity in organellar compared to nuclear genomes.

### 2.3 Sequence similarity between mitochondrial and chloroplast genomes

**Figure 3:**
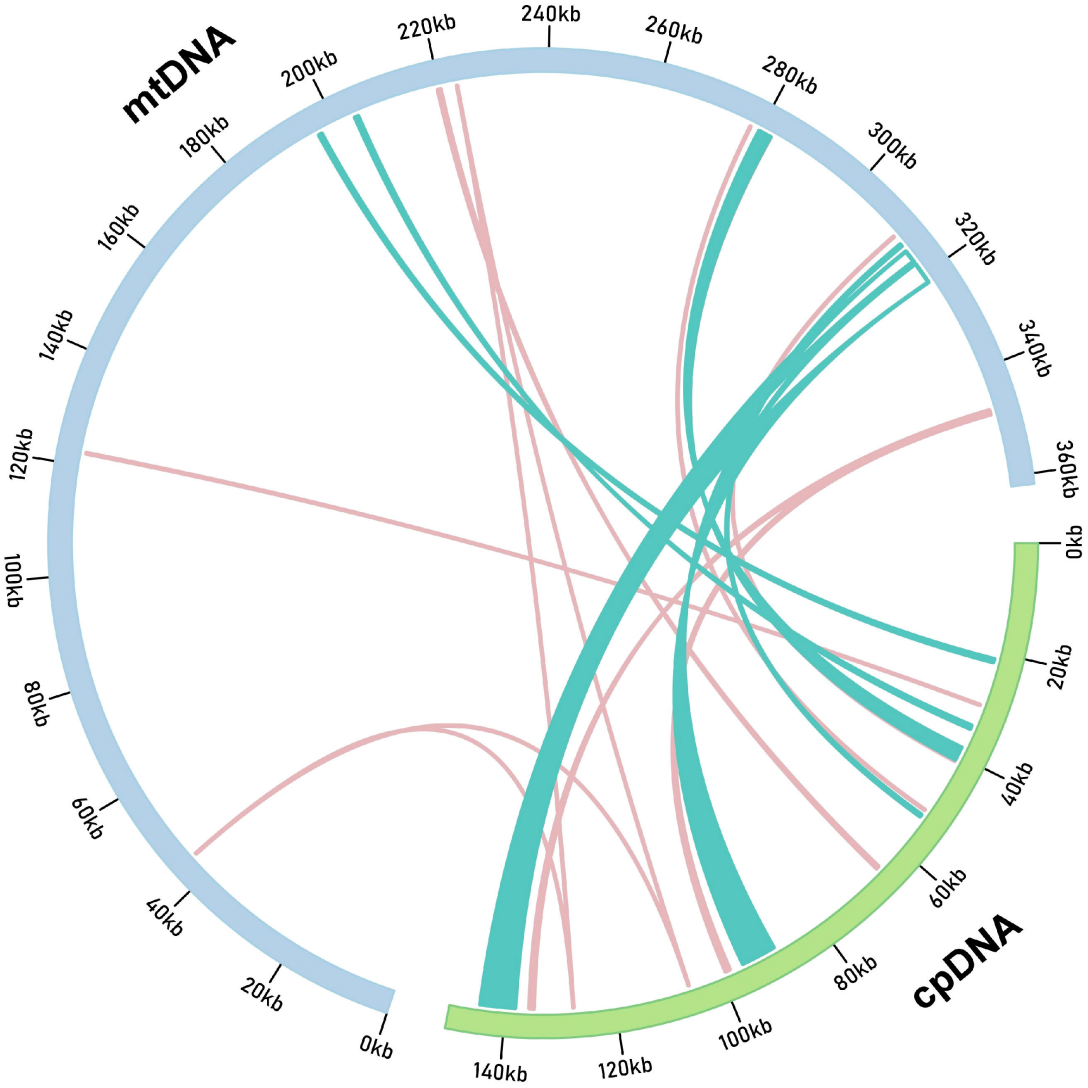
*G. vaccaria* of the transfer of chloroplast genes to the mitochondrial genome. The orange and teal blue lines within the circles represent the distribution of homologous genes on the positive and negative strands, respectively. (cpDNA: chloroplast genome; mtDNA: mitochondrial genome).

The mitochondrial genome of *G. vaccaria* spans 361,814 bp, representing a 2.41-fold size expansion compared to its chloroplast counterpart (150,050 bp). Comparative analysis identified 20 chloroplast-derived DNA (MTPT) fragments in the mitogenome through BLASTn alignment, collectively spanning 56,732 bp and constituting 15.6% of the mitochondrial genome (**Figure 3**; **Supplementary Table S9**). Notably, 12 chloroplast-derived genes retained intact ORFs in the mitogenome, including five protein-coding genes (*rps7*, *ndhB*, *psaB*, *petG*, *ycf15*), 6 tRNA genes (*trnL-CAA*, *trnW-CCA*, *trnP-UGG*, *trnN-GUU*, *trnD-GUC*, *trnM-CAU*), and 1 rRNA gene *(rrn16S*). Among these, *ndhB* gene-encoding a NADH dehydrogenase subunit critical for the electron transport chain—represented the longest intact MTPT fragment (757 bp), suggesting potential functional retention in the mitochondrial genome (**Figure 3**; **Supplementary Table S9**), while *trnM-CAU* was the shortest at 77 bp. The presence of these homologous fragments suggests possible genomic recombination or transfer of genetic material between the chloroplast and mitochondrial genomes.

### 2.4 Comparative analysis of codon usage bias between organellar genomes

**Figure 4:**
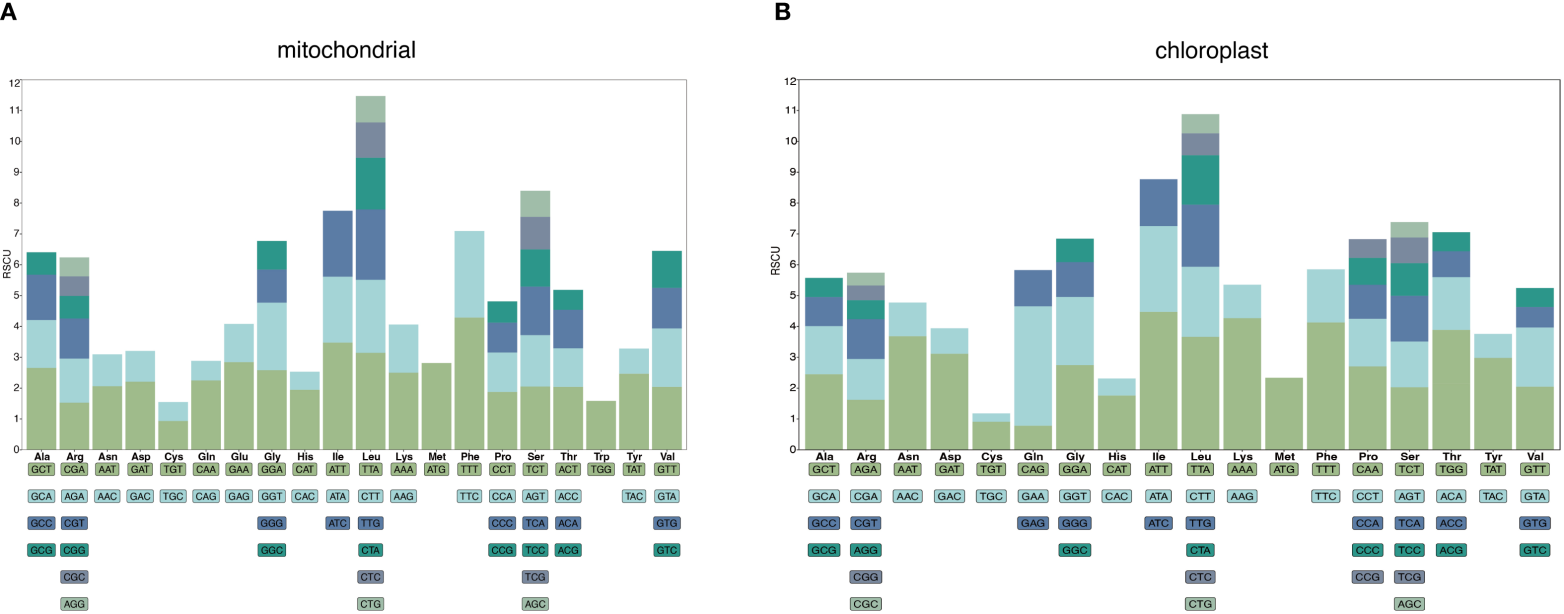
Analysis of relative synonymous codon usage (RSCU) in the *G vaccaria* mitochondrial **(A)** and chloroplast **(B)** genome. The following blocks represent all codons encoding each amino acid, and the height of the upper column represents the sum of RSCU values of all codons.

A total of 9,600 codons were identified in the *G. vaccaria* mitogenome. Among these codons, Phenylalanine (Phe, TTT codon) was the most frequently used amino acid, with a total of 412 codons, accounting for 4.29%, followed by isoleucine (Ile), with a total of 334 codons, accounting for 3.48%, and the lowest usage rate was termination codon, with a total of 22 codons, accounting for 0.23%. The AUG codon of methionine showed a high degree of preference in the *G. vaccaria* mt genome. Forty-five codons had RSCU values higher than one, indicating a preference for these codons. Among the 45 preferred codons, the third base of 29 codons ended with A or U, nine codons ended with G, and eight codons ended with C, which fully demonstrated the A/U preference of the third base of the codon (**Figure 4A**; **Supplementary Table S10**).

In contrast, the complete coding sequences of the *G. vaccaria* chloroplast genome comprised 23,984 codons. Among these codons, isoleucine (Ile, 1073 codons, 4.47%) was the most frequently encoded amino acid, followed by Lysine (Lys, 1024 codons, 4.27%), glutamine (Gln, 928 codons, 3.87%) and leucine (Leu, 879, 3.66%), while stop codons showed the lowest usage (82 codons, 0.34%). 39 codons had RSCU values higher than one, indicating a preference for these codons. Among the 39 preferred codons, the third base of 29 codons ended with A or U, six codons ended with G, and four codons ended with C, which fully demonstrated the A/U preference of the third base of the codon (**Figure 4B**; **Supplementary Table S10**).

### 2.5 Prediction of RNA editing sites in organellar genomes

**Figure 5:**
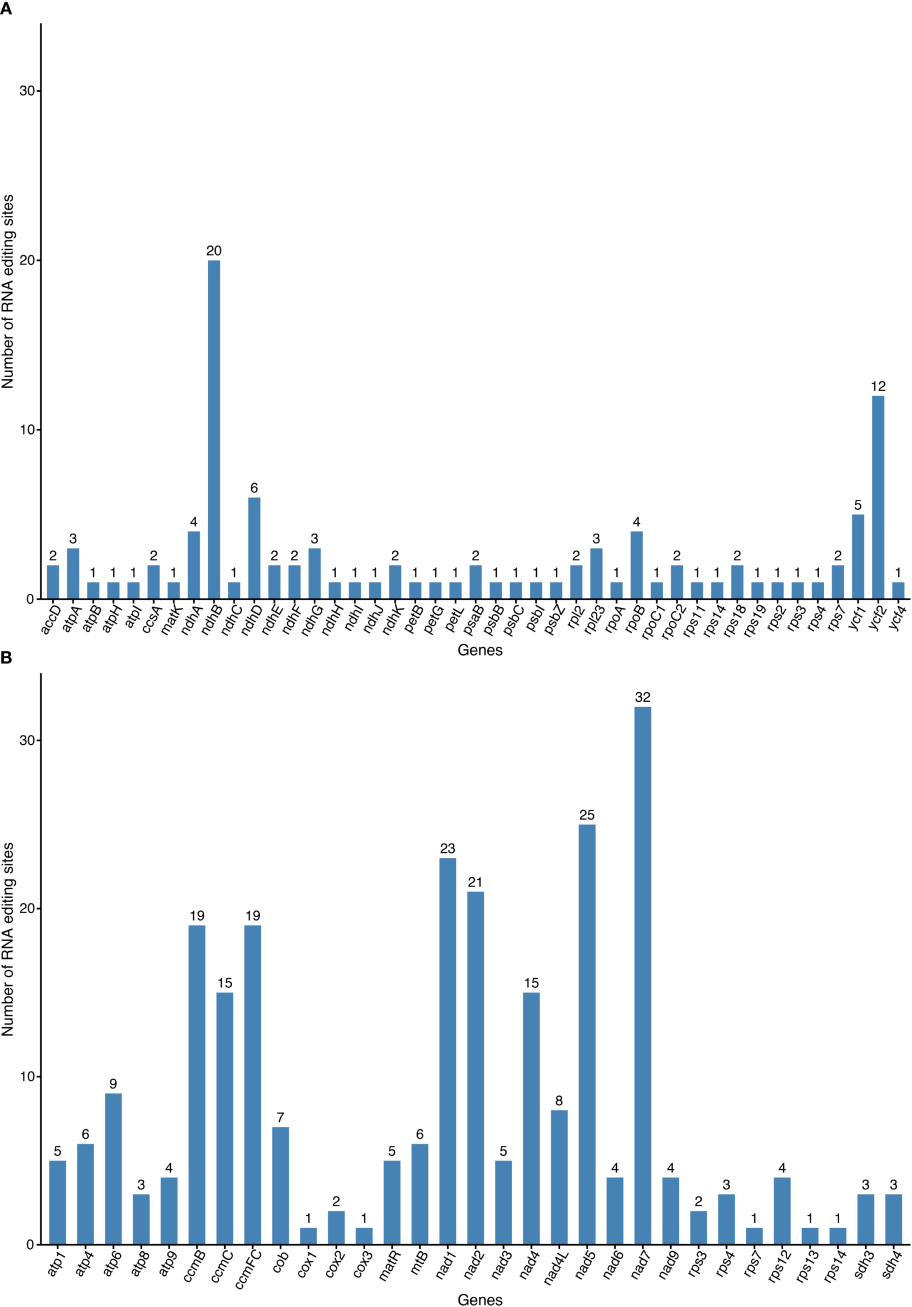
RNA editing events in the **(A)** mitochondrial and **(B)** chloroplast genomes of *G vaccaria*. The x-axis represents gene names, and the y-axis represents the number of RNA editing events.

We employed online prediction tools to analyze RNA editing patterns in 35 mitochondrial and 77 chloroplast protein-coding genes (PCGs). Analysis of the mitochondrial genome revealed 257 predicted RNA editing sites across all PCGs, exclusively involving C-to-U conversions (**Figure 5A**). The *nad7* (32 sites) genes exhibited the highest editing frequency, followed *nad5* (25 sites). Notably, certain genes, such as *cox1*, *cox3*, *rps7*, *rpsl3*, and *rpsl4*, exhibit only a single RNA editing event. In the chloroplast genome, 105 potential RNA editing sites were identified (**Figure 5B**). The *ndhB* (20 sites each) showed the highest editing activity, representing a 40.85% decrease compared to their mitochondrial counterparts. The *ycf2* genes ranked second with 12 editing sites. Among them, more than half of the genes (23/43) undergo only one RNA editing event. These findings demonstrate conserved RNA editing distribution patterns between mitochondrial and chloroplast genomes in *G. vaccaria*, while revealing organelle-specific differences in editing site abundance for homologous genes.

### 2.6 Ka/Ks analysis and phylogenetic analysis

**Figure 6:**
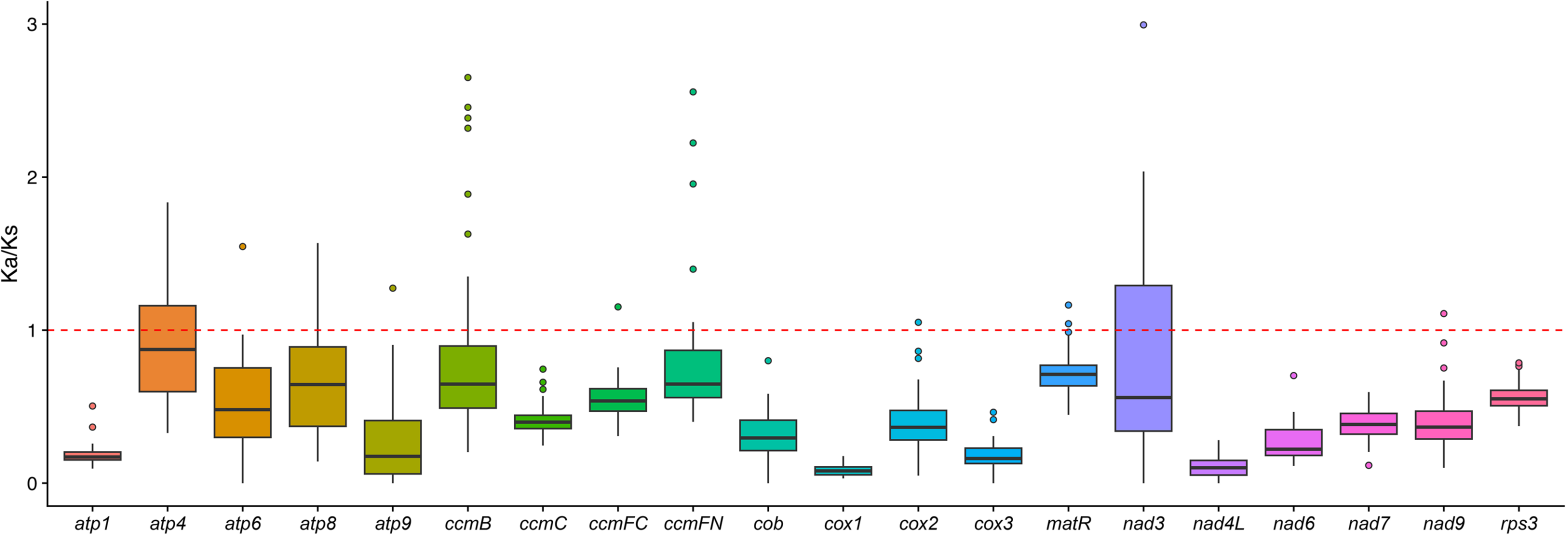
Ka/Ks ratio of *G. vaccaria* and eight other Caryophyllales plant species.

Evolutionary selection patterns were assessed through Ka/Ks (ω) ratio analysis of 20 conserved mitochondrial genes across nine Caryophyllales species. The median ω value across all genes was 0.32 (IQR:0.18-0.47), indicating predominant purifying selection (**Figure 6**). Three cytochrome c maturation genes showed signatures of positive selection (ω>1) in Caryophyllaceae.

In our study, we calculated the Ka/Ks ratios for the shared genes in the mitochondrial genomes of nine Caryophyllales species. The median Ka/Ks value for all 20 shared genes was found to be less than 1, indicating that these genes have undergone purifying selection during the evolutionary process of Caryophyllales species. This suggests that these genes are evolutionarily conserved.

Further comparisons of Ka/Ks ratios between *G. vaccaria* (a Caryophyllaceae species) and two other Caryophyllales species revealed that the Ka/Ks ratio of the *ccmFN* gene was greater than 1 in *G. vaccaria*, indicating that this gene underwent positive selection within the Caryophyllaceae family. Similarly, the *ccmB* and *ccmFC* genes also showed Ka/Ks ratios greater than 1 in the comparison between *G. vaccaria* and *Agrostemma githago*, suggesting that these two genes experienced positive selection during the evolution of the two species. Additionally, the Ka/Ks ratios of the *atp4*, *matR*, and *nad3* genes in *G. vaccaria* were greater than 1 when compared to *Rheum palmatum*, indicating that these three genes underwent positive selection in both species.

**Figure 7:**
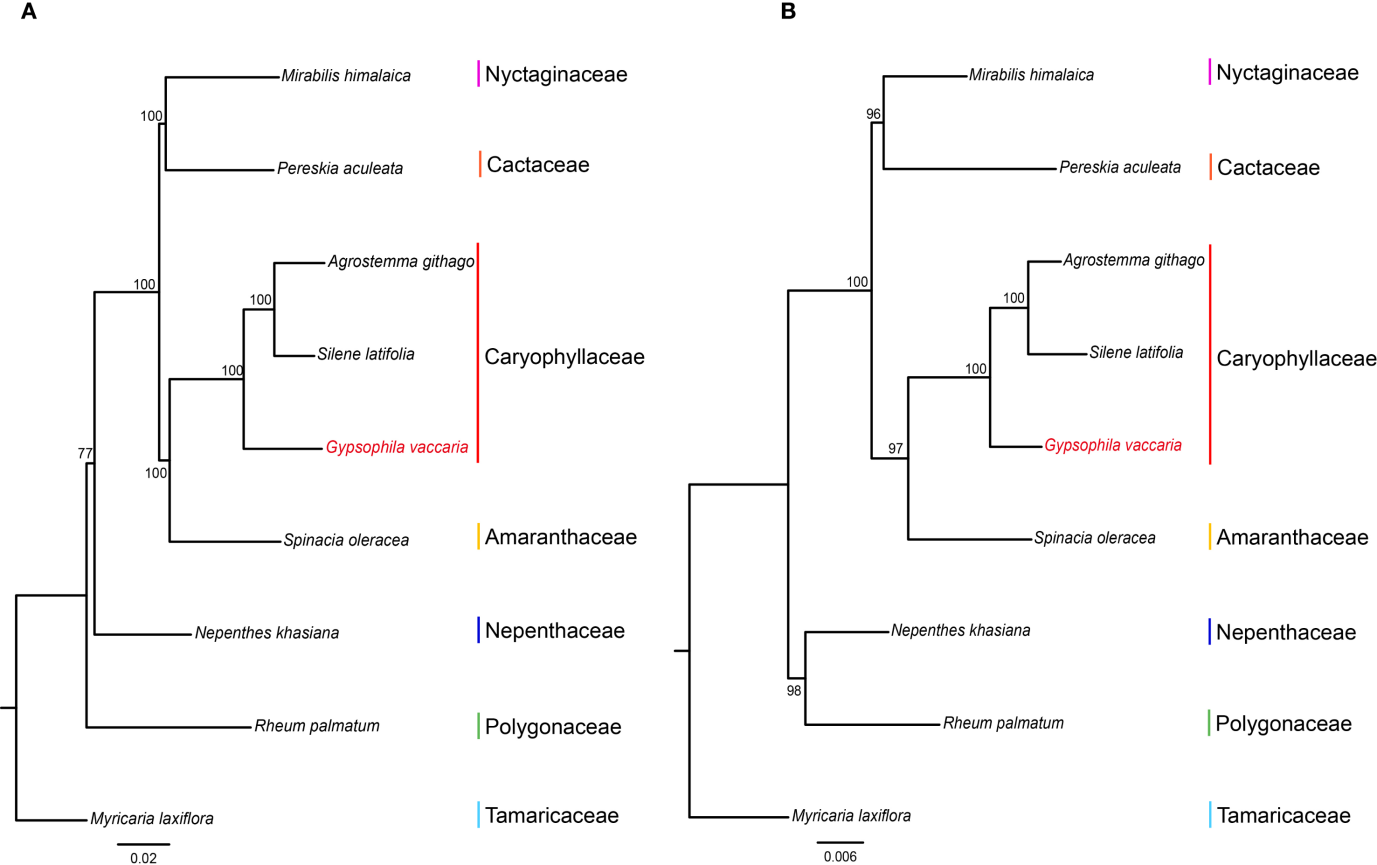
Phylogenetic relationships of *G vaccaria* and eight Caryophyllales species based on shared genes of mitochondrial genome and chloroplast genome. **(A)** A phylogenetic tree constructed by Maximum likelihood (ML) based on shared genes of 9 chloroplast genomes. **(B)** A phylogenetic tree constructed by Maximum likelihood (ML) based on shared genes of 9 mitochondrial genomes.

A phylogenetic analysis was conducted on the mitochondrial and chloroplast genomes of two species from the Caryophyllaceae family, along with six species from six different families in the order Caryophyllales, including *Dianthus* (carnation). The species *Myricaria laxiflora* from the Tamaricaceae family was used as the outgroup (**Figure 7**). The phylogenetic trees constructed from both mitochondrial and chloroplast genomes showed that the three Caryophyllaceae species clustered together as a single clade. In addition, the Caryophyllaceae family initially grouped with Amaranthaceae, followed by a further clustering with Nyctaginaceae and Cactaceae. In the mitochondrial tree, Nepenthaceae and Polygonaceae formed a distinct clade, although in the chloroplast tree, these two families did not cluster together. Overall, our phylogenetic analysis of Caryophyllales species with publicly available mitochondrial and plastid genomes from NCBI supports a close relationship between the Caryophyllaceae family and the Amaranthaceae family, which is consistent with previous studies.

## 3 Discussion

Mitochondria drive ATP synthesis in plant cells, critically regulating growth and development (Mackenzie and McIntosh, 1999). Recent comparative studies revealed that the evolutionary trajectories of mitogenomes exhibit striking divergence, leading to extensive structural variations among plant lineages. For example, while *Cinnamomum chekiangense*, and *Salix truncatum* maintain a canonical circular mitochondrial genome (Han et al., 2022; Bi et al., 2024), other species exhibit structural diversity—*Abelmoschus esculentus* harbors two distinct configurations (Li et al., 2022), and *Sorghum bicolor* and *Populus simonii* demonstrate three or more structural isoforms (Bi et al., 2022; Zhang et al., 2023a). In this study, using high-fidelity PacBio HiFi long-read sequencing data, we assembled and annotated the complete mitochondrial (361,814 bp) and chloroplast (150,050 bp) genomes of *G. vaccaria* (Caryophyllaceae). The chloroplast genome exhibits a canonical quadripartite structure with conserved gene content (122 genes). Comparative analysis revealed that the chloroplast genome size, GC content, and gene number of *G. vaccaria* are similar to those of other reported Caryophyllales species (e.g., *Agrostemma* and *Silene*), where the inverted repeat (IR) regions may stabilize the genomic structure by preventing recombination-induced gene loss. In contrast, the mitochondrial genome exhibits a closed circular structure with 2.4-fold size expansion relative to chloroplast genomes. This expansion aligns with a characteristic trend observed in Caryophyllaceae (e.g., *Silene*), primarily driven by proliferated repetitive elements (e.g., SSRs, TRs; accounting for 88.2% of the mitogenome) and large-scale chloroplast-to- mitochondrion DNA transfers (MTPTs; spanning 56,732 Kb, 15.6% of the mitogenome), as resolved unambiguously by HiFi reads.

To place these findings into a broader evolutionary context, we compared the *G. vaccaria* mitogenome with those of related taxa within Caryophyllales. Within the order Caryophyllales, substantial variation in mitogenome size and structure (circular/multipartite) has been reported (Gao et al., 2023). While angiosperm mitogenomes typically range between 200–750 Kb (Burger et al., 2003; Gualberto and Newton, 2017), extreme cases include highly reduced (<100 Kb; *Viscum scurruloideum*) (Gao et al., 2023) or expanded (>11 Mb; *Silene conica*) (Gualberto and Newton, 2017) genomes. Within the Caryophyllaceae family, the mitogenome of *G. vaccaria* (361,814 bp) is notably larger than those of its nonfamilial relatives *Agrostemma githago* (262,903 bp) (Gao et al., 2023) and *Silene latifolia* (253,413 bp) (Sloan et al., 2010). However, when compared to other families within Caryophyllales, its size aligns with *Mirabilis himalaica* (Nyctaginaceae;346,363 bp) (Yuan et al., 2020), *Spinacia oleracea* (Amaranthaceae; 329,613 bp) (Cai et al., 2017), *Rheum palmatum* (Polygonaceae; 302,993 bp) (Gao et al., 2023), and *Myricaria laxiflora* (Tamaricaceae; 389,949 bp) (Wang et al., 2024). In contrast, it remains smaller than the mitogenomes of *Pereskia aculeata* (Cactaceae; 515,187 bp) (Zhang et al., 2023b) and *Nepenthes khasiana* (Nepenthaceae; 900,031 bp) (Konhar et al., 2025). Notably, extreme expansions occur in some Caryophyllaceae species, such as *Silene noctiflora* (6.7–7.1 Mb) (Wu et al., 2015) and *Silene conica* (11.3 Mb) (Smith, 2020), highlighting family-specific genomic plasticity (**Supplementary Table S11**). Despite these expansions, 99% of the increased genome size in large mitogenomes consists of intergenic sequences, with only ~1% derived from chloroplast or nuclear DNA (Sloan et al., 2012a; Gualberto and Newton, 2017). The origins of the remaining intergenic regions, potentially involving *de novo* repeats or horizontal transfers, remain unresolved. While recent studies suggested that genome-size circular molecules are rare in mitogenomes, with multi-isoform structures being more prevalent (Smith and Keeling, 2015), our analysis of 27 Caryophyllales mitogenomes revealed that 41.46% (17/41) were assembled as single circular maps. However, 41.18% (7/17) of these single circular assemblies relied on Illumina short-read sequencing (<300 bp), which has limited power to resolve complex structural variants such as alternative conformations or recombination-derived isoforms (Kozik et al., 2019) (**Supplementary Table S11**). During the assembly of the *G. vaccaria* mitochondrial genome, we carefully considered the possibility that the observed circular conformation might represent an assembly artifact. Read-mapping depth and HiFi coverage analyses revealed consistent support across the genome, suggesting that the circular form is likely genuine. Although PCR validation of the circular junctions was not performed in this study, future work could experimentally confirm genome circularity and reconcile potential discrepancies with earlier studies that may have overestimated single circular mitogenomes.

In addition, the GC content of the *G. vaccaria* mitogenome (43.82%) falls within the range of other Caryophyllales species (42.6–45.23%, **Supplementary Table S11**), Specifically, values for Caryophyllaceae species are 44.7% (*Agrostemma githago*) and 42.6% (*Silene latifolia*), while non-Caryophyllaceae members exhibit 43.4-45.23 (e.g., *Spinacia oleracea*, *Rheum palmatum*), suggesting family-level GC homogeneity. This study represents the first mitogenomic characterization of the genus *Gypsophila*, providing a foundation for exploring evolutionary mechanisms in Caryophyllaceae.

Plant mitogenomes frequently harbor plastid-derived sequences (MTPTs), with the extent of plastid-to-mitochondrion DNA transfer exhibiting significant interspecific variation (Wang et al., 2018). Comparative analyses have demonstrated that the total length of MTPTs can range from <1 Kb in *Vigna angularis* to >130 Kb in *Amborella trichopoda* (Sloan and Wu, 2014), suggesting lineage-specific dynamics of inter-organellar gene transfer. In this study, the total length of MTPTs in *G. vaccaria* was 56,732 bp, accounting for 15.69% of the entire mitogenome. This proportion markedly exceeds the 1-12% range reported in most angiosperms (Kubo and Mikami, 2007; Alverson et al., 2010), suggesting exceptional frequency of plastid-to-mitochondrion DNA transfer in *G. vaccaria*. Comparative analysis revealed divergent patterns of chloroplast-to-mitochondrion DNA transfer, with 12 out of 20 chloroplast-derived homologous sequences retaining intact functional genes—five protein-coding genes (e.g., *rps7*, *ndhB*), six tRNAs (e.g., *trnL-CAA*, *trnW-CCA*), and one rRNA (*rrn16S*). These MTPTs likely originated via retrotransposition or double-strand break repair, consistent with unidirectional transfer patterns observed in *Silene*. Notably, the *ndhB* (757 bp) and *rrn16S* genes retained full-length ORFs, raising questions about their functionality in the mitochondrial context. The exclusive presence of forward repeats (0 inverted repeats) in MTPTs implies unidirectional integration via non-homologous end joining (NHEJ), contrasting with recombination-driven plastid DNA incorporation in Silene (Smith, 2020). This Caryophyllaceae-specific mechanism may explain the exceptional MTPT accumulation (15.6% vs. 1-12% in most angiosperms).

Beyond protein-coding genes, tRNA gene sequences showing high similarity between the mitogenome and chloroplast genome were considered to be plastid-derived sequences. The *trnH-GUG*, *trnM-CAU*, *trnN-GUU*, *trnW-CCA*, *trnP-UGG*, and *trnS-GGA* are common plastid-derived genes in angiosperm, *trnD-GUC* is common only in dicots (Sugiyama et al., 2005). In *G. vaccaria*, we annotated five plastid-derived tRNAs (*trnH-GUG*, *trnW-CCA*, *trnN-GUU*, *trnM-CAU*, and *trnP-UGG*) within MTPTs, but *trnS-GGA* was absent. tRNA transfer events provide additional insights into MTPT dynamics, revealing both conserved transfer pathways and lineage-specific gene loss patterns in Caryophyllaceae. Although some MTPTs retain homologies to PVGs (plastid-derived viral genes) and functional tRNAs, empirical studies demonstrate their progressive degradation post-transfer (Notsu et al., 2002; Ogihara et al., 2005; Wang et al., 2007), rendering them non-functional in mitochondria. Our identification of MTPTs in *G. vaccaria* will facilitate the accurate assembly of chloroplast and mitochondrial genomes, and enhance our understanding of organelle genome evolution.

Codon usage bias refers to the phenomenon in which certain codons are used more frequently than others in the DNA or RNA sequences of specific organisms, often associated with gene expression efficiency and protein assembly processes (Sloan et al., 2012a; Smith and Keeling, 2015). Significant differences in codon usage preferences are observed between different species, and even between different organelles within the same species. To investigate the codon usage bias and its differences between the chloroplast and mitochondrial genomes of *G. vaccaria*, we calculated the relative synonymous codon usage (RSCU) values for each. Codons with RSCU values greater than 1 indicate a preference for the corresponding amino acid. Mitochondrial genes exhibited pronounced A/U-ending codon preference (29/44 high-RSCU codons), correlating with low GC content (43.8%) and likely reflecting mutational bias. Phenylalanine (TTT) and isoleucine were the most frequent amino acids, possibly linked to elevated demand for respiratory chain proteins. Chloroplast genes showed similar but weaker A/U bias, implying convergent translational optimization across organelles. HiFi sequencing resolved codon-level patterns with high accuracy, eliminating ambiguities from homopolymer errors common in short-read data. RNA editing, a ubiquitous post-transcriptional modification in plant organelles, fine-tunes mitochondrial gene expression by introducing nucleotide substitutions (e.g., C-to-U conversions), thereby regulating crucial physiological processes such as energy metabolism and stress responses (Small et al., 2020). We predicted 257 C-to-U RNA editing sites in mitochondrial PCGs—far exceeding the 105 sites in chloroplasts—with *nad7* (32 sites) and *nad5* (25 sites) being hotspots. This divergence may reflect stricter functional constraints on mitochondrial electron transport chain genes. Chloroplast *ndhB* (20 sites) and *ycf2* (12 sites) showed reduced editing activity (-40.8% vs. mitochondrial), possibly due to relaxed selection or alternative regulatory mechanisms. HiFi data enabled precise mapping of editing sites, avoiding false positives from misaligned short reads. The strong positive selection on cytochrome c maturation genes (*ccmFN/B/FC*; ω=1.2-1.5) may reflect adaptations to arid environments, as *ccm* complexes regulate heme biosynthesis critical for stress-responsive hemoproteins (Stevens et al., 2011).

Beyond genomic characterization, our study provides high-quality mitochondrial and chloroplast genome assemblies and 81 species-specific SSR markers for *G. vaccaria*, offering reliable molecular tools for cultivar identification, parent selection, and marker-assisted breeding. Organelle-derived SSRs have proven effective in species discrimination and genetic diversity analyses in other medicinal and crop plants (Li et al., 2025), highlighting their potential to accelerate breeding and track desirable traits. The integration of plastid DNA into the mitogenome in *G. vaccaria*, likely mediated by unidirectional non-homologous end joining (NHEJ), appears to differ from the recombination-driven incorporation reported in Silene (Sloan et al., 2012b; Wu et al., 2015), implying that Caryophyllaceae may exhibit distinct mechanisms influencing organelle genome evolution.

Additionally, the expansion of the mitochondrial genome may be partly influenced by nuclear-integrated mitochondrial sequences (NUMTs). Although a systematic analysis of NUMTs was beyond the scope of this study, previous research suggests that their insertions can contribute to increased genome size and structural complexity (Kleine et al., 2009). In the present study, we did not perform a systematic analysis of NUMTs in *G. vaccaria*, and therefore the potential contribution of NUMTs to mitochondrial–nuclear genome interactions remain unexplored. Future research could employ genome-wide comparative approaches to identify NUMTs, assess their abundance and insertional patterns, and evaluate their potential functional or evolutionary implications in *G. vaccaria*. Such investigations would provide deeper insights into the dynamics of intracellular DNA transfer and their role in shaping genome architecture in this species, thereby enhancing our understanding of plant genome complexity and evolution.

Collectively, these findings not only advance our understanding of Caryophyllaceae mitogenome evolution but also establish a foundation for molecular breeding, functional genomics, and stress adaptation studies in this medicinally important species.

## 4 Materials and methods

### 4.1 Plant materials, DNA extraction, and sequencing

Fresh leaves from the same *Gypsophila vaccaria* plant were collected at the Medicinal Plant Germplasm College of Hexi University (100.441871°E, 38.948448° N; 1,256 m). The leaves were carefully cleaned with DEPC water and stored at -80 °C in the key laboratory of Hexi Corridor Resources Utilization of Gansu, Hexi University. High-quality genomic DNA was extracted using a DNA plant extraction kit (Tiangen, China). The DNA quality was evaluated with agarose gel electrophoresis, and its concentration was measured using a Nanodrop instrument (Thermo Fisher Scientific, Waltham, MA, USA). The qualified DNA samples were subsequently sent to Beijing BerryGenomics Co., Ltd. for Illumina sequencing and high fidelity (HiFi) sequencing.

### 4.2 Organelle genome assembly and annotation

The chloroplast and mitochondrial genomes of *G. vaccaria* were *de novo* assembled using Oatk v1.0 with default parameters based on HiFi reads (Zhou et al., 2025). The assembly results were visualized and manually curated in Bandage v0.8.1 with default parameters to generate fasta format files (Wick et al., 2015). To offer a more accurate view of the genome assembly, these two organelle genomes were mapped back HiFi reads to calculate the sequencing coverage depth using minimap2 v2.26 and samtools v1.17 with default parameters (Li and Liang, 2007; Li, 2018).

The chloroplast genome of *G. vaccaria* was annotated using CPGAVAS2 (Shi et al., 2019), GeSeq (Tillich et al., 2017) and CPGview (Liu et al., 2023), while the mitochondrial genome was annotated using IPMGA (http://www.1kmpg.cn/ipmga/), GeSeq (Tillich et al., 2017) and CPGview with default parameters (Liu et al., 2023). The tRNA genes were annotated using tRNAscan-SE v2.0.7 (Chan et al., 2021), and the open reading frames (ORFs) were annotated using the Open Reading Frame Finder (Rombel et al., 2002). During the annotation process, sequences overlapped with known genes were excluded, and sequences longer than 300 bp were further aligned against the NR database for additional functional annotation. To ensure the accuracy of the annotation results, all data were manually inspected and corrected. Additionally, the visualization of the chloroplast and mitochondrial genomes was automatically generated by CPGAVAS2 (Shi et al., 2019) and IPMGA, respectively.

### 4.3 Analysis of repeated sequences

The repeat sequence analysis includes microsatellite repeats (also known as simple sequence repeats, SSRs), tandem sequence repeats (TSRs), and dispersed sequence repeats (DSRs). In this study, microsatellite repeats were identified using the Misa-web tool (Beier et al., 2017). Tandem repeats (with lengths >6 bp) were detected using Tandem Repeats Finder with default parameters (Benson, 1999). Dispersed repeats were analyzed through the BiBiserv2 platform(https://bibiserv.cebitec.uni-bielefeld.de/). Finally, the identified repeat sequences were visualized using the Circos package in TBtools v2.056 (Chen et al., 2020).

### 4.4 DNA transfer between the chloroplast and the mitochondrion

To detect potential DNA transfers between the chloroplast genome and the mitochondrial genome of *G. vaccaria*, BLASTN was used for sequence similarity analysis with an e-value threshold of 1e-5 (Camacho et al., 2009). This analysis identified potential regions of DNA fragment transfer. Finally, the results were visualized using the Circos package in TBtools v2.056 (Chen et al., 2020).

### 4.5 Codon usage and RNA editing analysis

To explore the combined effects of natural selection, mutation, and genetic drift on codon usage, this study analyzed the relative synonymous codon usage (RSCU). A custom Perl script was used to perform a detailed analysis of the codon composition in the chloroplast and mitochondrial genomes of *G. vaccaria*. The analysis included the identification of unique coding sequences (CDS), determination of the codon counts for each gene, calculation of the effective number of codons (Nc), and further comparison of the RSCU values of synonymous codons between the chloroplast and mitochondrial genomes.

In this study, plant mitochondrial protein-coding genes were used as reference sequences, and the PREPACT v3.12.0 with default parameters was employed to identify RNA editing sites in the chloroplast and mitochondrial RNAs of *G. vaccaria* (Lenz et al., 2018).

### 4.6 Phylogenetic and Ka/Ks analysis

To determine the phylogenetic relationship of *G. vaccaria*, eight sequenced chloroplast and mitochondrial genomes of Caryophyllales species, including *Myricaria laxiflora, Rheum palmatum, Nepenthes khasiana, Spinacia oleracea, Silene latifolia, Agrostemma githago, Mirabilis himalaica, Pereskia aculeata* were downloaded from NCBI database (**Supplementary Table S12**). Phylogenetic trees were conducted based on shared chloroplast and mitochondrial genes among these 9 species including *G. vaccaria*. The shared chloroplast and mitochondrial gene CDS sequences were aligned using MAFFT (Lenz et al., 2018). Phylogenetic trees were constructed by RAxML with GTRGAMMA model and 1000 bootstraps (Stamatakis, 2014). Synonymous and nonsynonymous substitution ratios (Ka/Ks) were calculated by KaKs_Calculator v 3.0 (Zhang, 2022).

## 5 Conclusions

We present the first complete chloroplast and mitochondrial genomes of *Gypsophila vaccaria*, revealing key evolutionary features. The chloroplast genome (quadripartite structure) contains 77 PCGs, 37 tRNAs, and 8 rRNAs, while the mitochondrial genome (circular, 361,814 bp) harbors 35 PCGs, 21 tRNAs, and 3 rRNAs. Both organelles exhibit strong A/U codon bias (mitochondrial GC 44.2% vs. chloroplast 36.8%). Phylogenomic analyses resolve Caryophyllaceae-Amaranthaceae divergence (BS = 100%) and identify 81 species-specific SSRs. Positively selected sites in *ccmFN*, *ccmB*, *ccmFC* (ω=1.2-1.5, P<0.05) contrast with genome-wide purifying selection (median ω=0.32), suggesting adaptive evolution in cytochrome c maturation. Mitochondrial RNA editing (257 sites, e.g., 32 in *nad7*) exceeds chloroplast activity (105 sites), indicating divergent post-transcriptional regulation. Strikingly, 56.7 Kb of plastid-derived DNA (15.7% of mitogenome), including intact *rps7* and *ndhB*, underscores mitochondrial genome plasticity. These high-quality resources facilitate future studies on Caryophyllaceae evolution and biotechnology applications.

## Data Availability

The complete mitochondrial and chloroplast genome sequences were deposited in the NCBI GenBank (https://www.ncbi.nlm.nih.gov/genbank) under accession numbers PV700085 and PV700086. All supporting data are included in **Supplementary Table 1**.
